# The Ancestral Eutherian Karyotype Is Present in Xenarthra

**DOI:** 10.1371/journal.pgen.0020109

**Published:** 2006-07-21

**Authors:** Marta Svartman, Gary Stone, Roscoe Stanyon

**Affiliations:** Comparative Molecular Cytogenetics Core, Genetics Branch, National Cancer Institute-Frederick, Frederick, Maryland, United States of America; Fred Hutchinson Cancer Research Center, United States of America

## Abstract

Molecular studies have led recently to the proposal of a new super-ordinal arrangement of the 18 extant Eutherian orders. From the four proposed super-orders, Afrotheria and Xenarthra were considered the most basal. Chromosome-painting studies with human probes in these two mammalian groups are thus key in the quest to establish the ancestral Eutherian karyotype. Although a reasonable amount of chromosome-painting data with human probes have already been obtained for Afrotheria, no Xenarthra species has been thoroughly analyzed with this approach. We hybridized human chromosome probes to metaphases of species *(Dasypus novemcinctus, Tamandua tetradactyla,* and *Choloepus hoffmanii)* representing three of the four Xenarthra families*.* Our data allowed us to review the current hypotheses for the ancestral Eutherian karyotype, which range from 2*n* = 44 to 2*n* = 48. One of the species studied, the two-toed sloth C. hoffmanii (2*n* = 50), showed a chromosome complement strikingly similar to the proposed 2*n* = 48 ancestral Eutherian karyotype, strongly reinforcing it.

## Synopsis

Living mammals are classified into three major groups: monotremes, marsupials, and placental mammals or Eutherians, composed of 18 orders. Phylogenetic studies point to Afrotheria (a clade of six endemic African orders) or Xenarthra (armadillos, anteaters, and sloths, mostly found in Central and South America) as the most basal Eutherian group. One of the most daring aims of molecular cytogenetics in the past decade has been to establish the karyotype present in a common ancestor of all Eutherians. The approach used, cross-species chromosome painting, involves the use of probes from specific chromosomes or chromosome segments from one species, which are hybridized to metaphases of another species, highlighting regions of homology between both genomes. More than 60 species have already been analyzed with this method, and it is believed that the ancestral karyotype had 44, 46, or 48 chromosomes. The authors, using human chromosomes as probes to study three Xenarthra species, found that one of them, the two-toed sloth Choloepus hoffmannii (2*n* = 50), has a karyotype strikingly similar to the proposed 2*n* = 48 ancestral Eutherian complement. This observation, besides reinforcing the hypothesized karyotype, suggests that Xenarthra may be at the root of the Eutherian tree.

## Introduction

Extensive molecular data on mammalian genomes have led recently to the proposal of a new phylogenetic tree for Eutherians, which encompasses four super-orders: Afrotheria (elephants, manatees, hyraxes, tenrecs, aardvark, and elephant shrews); Xenarthra (sloths, anteaters, and armadillos); Euarchontoglires (rodents and lagomorphs as a sister taxon to primates, flying lemurs, and tree shrews); and Laurasiatheria (cetaceans, artiodactyls, perissodactyls, carnivores, pangolins, bats, and insectivores) [1,2]. At first very debated, the grouping of the 18 living placental mammalian orders into these four clades is rapidly approaching a consensus [3]. One of the pivotal questions still remaining relates to the root of the placental tree. Either Xenarthra or Afrotheria, or the combination Xenarthra plus Afrotheria, could represent the first split from a common Eutherian ancestor (reviewed in [3]). Although Afrotheria has been favored as the first offshoot in the Eutherian tree [2], both Xenarthra and Afrotheria emerged around 100 million years ago (mya), within a short interval from each other [4,5]. This is also the time of the separation between South America, where Xenarthrans are endemic, and Africa, home to the Afrotheria. Therefore, a causal connection between the plate-tectonic events and the diversification of mammals has been proposed [1,3].

Extant Xenarthra (sloths, anteaters, and armadillos) are restricted to the Americas, mostly Central and South America. The 30 known living species are relicts of the once highly diversified Xenarthra that dominated the South American fauna from the late Cretaceous (80–65 mya) until the late Tertiary (3–2.5 mya), while South America was an isolated landmass. Xenarthrans were abundant until the Pleistocene (10,000 years ago), and more than 200 fossil genera have been reported [5].

Morphological and molecular studies support the monophyly of the group, which also extends to each of the four recognized extant families: Myrmecophagidae (four species of anteater), Bradypodidae (four species of three-toed sloth), Megalonychidae (two species of two-toed sloth), and Dasypodidae (about 20 species of armadillo) [6–9]. According to molecular data estimates, the radiation of Xenarthra occurred around 65 mya, during the Cretaceous/Tertiary boundary. Armadillos, the most basal group of Xenarthra, diverged around 65 mya, followed by the divergence between anteaters and sloths around 55 mya [5].

Molecular data on Xenarthra are rapidly accumulating in the literature [5–9], but only scarce information is available on their chromosomes. Nineteen species had their karyotypes described [10], but only a few with banding techniques [11–13]. The reported diploid numbers range from 2*n* = 38 in Tolypeutes matacus to 2*n* = 64 in *Dasypus novemcinctus, D*. *hybridus,* and Cyclopes didactyla [11]. The first comparative study between karyotypes of different Xenarthra species using molecular cytogenetics has recently been published, and the rate of chromosome repatterning in Xenarthra was considered low [13].

Chromosome painting to establish the karyotype of a common ancestral Eutherian mammal has already been applied in 12 out of the 18 orders in the group. The proposed diploid numbers range from 2*n* = 44 to 2*n* = 50 [14–21]. The most recent studies suggest 2*n* = 44 [19], 2*n* = 46 [20], and 2*n* = 48 [17,21]. Chromosome-painting studies in Afrotheria and Xenarthra, the most basal of the living Eutherians, are essential to establish the putative ancestral Eutherian complement. Some Afrotheria have already had their genomes analyzed by chromosome painting with human probes [19–22]. Data of comparative molecular cytogenetics in Xenarthra using human chromosomes as painting probes are restricted to very limited data for two species [17,18,23].

In this study, we hybridized paints of all human chromosomes in species representing three of the four families of Xenarthra, and reevaluated the current hypotheses for the ancestral Eutherian karyotype based on our results.

## Results

Some examples of human chromosome paints hybridized to the three Xenarthra species are shown in Figure 1. The results of the chromosome painting with human probes in the three species are summarized in Table 1. The G-banded karyotype of the three species studied were mounted according to Jorge et al. [11] and are presented with the corresponding human chromosome segments in Figure 2 *(D. novemcinctus* and *Tamandua tetradactyla)* and Figure 3A *(Choloepus hoffmanii).*

**Figure 1:**
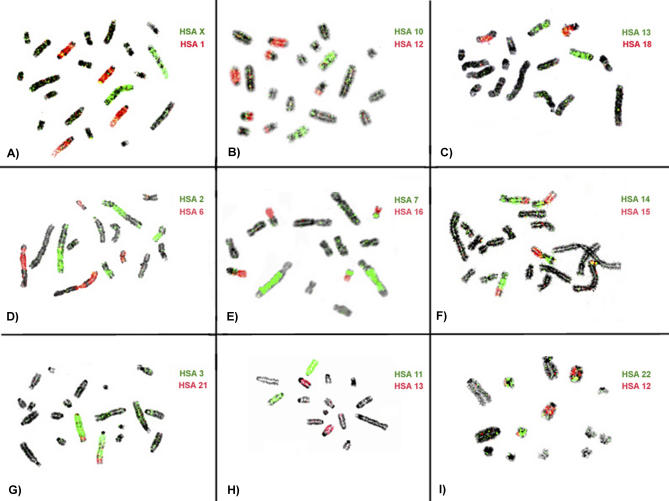
Partial Metaphases of *D. novemcinctus, T. tetradactyla,* and C. hoffmanii after In Situ Hybridization with Human Chromosome–Specific Probes (A–C) *D. novemcinctus,* (D–F) *T. tetradactyla,* and (G–I) C. hoffmanii. Two probes were used in each experiment, and the hybridizations were detected with avidin–FITC (green) and antidigoxigenin–rhodamine (red). The chromosomes were counterstained with DAPI, and the human chromosome probes used are indicated.

**Table 1:**
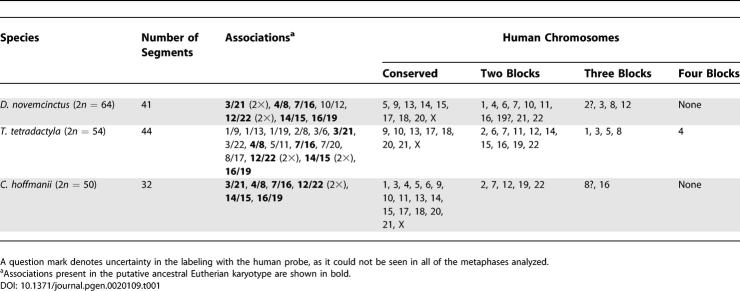
Results of Chromosome Painting with Human Probes in Xenarthra

The X chromosome of the three Xenarthra species hybridized with human chromosome X and showed a highly conserved G-banding pattern when compared to the human X chromosome, reinforcing its known conservation. No signals were obtained with human chromosome Y.

In the nine-banded armadillo D. novemcinctus (2*n* = 64; fundamental number [*FN*] = 78), chromosome painting resulted in 41 segments, and seven associations, or combinations of segments from two human chromosomes, were observed (Figure 2A).

**Figure 2:**
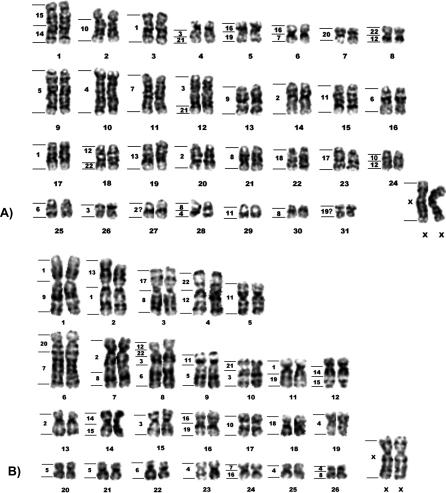
G-Banded Karyotypes of Female Nine-Banded Armadillo and Female Lesser Anteater (A) Female nine-banded armadillo D. novemcinctus (2*n* = 64) and (B) female lesser anteater T. tetradactyla (2*n* = 54). The correspondence to human chromosomes as revealed by chromosome painting is shown on the left of each chromosome. The question mark indicates questionable results. Some regions were not painted by any human probe and are probably composed of repetitive sequences (see text).

The karyotype of the lesser anteater T. tetradactyla (2*n* = 54; *FN* = 104) consists exclusively of biarmed chromosomes. Painting with human probes resulted in a total of 44 segments, and 15 associations were observed (Figure 2B).

The two-toed sloth C. hoffmanii is the species with the lowest diploid number (2*n* = 50; *FN* = 61). Owing to a heterozygous translocation between pairs 2 and 24, pair 2 was heteromorphic in the cells analyzed; this individual is thus trisomic for Chromosome 24. A total of 32 segments and six associations were detected with the human probes (Figure 3A).

**Figure 3:**
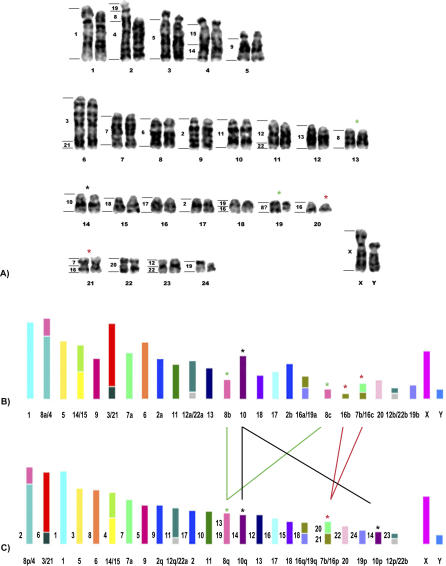
The Karyotype of Hoffmann's Two-Toed Sloth (2*n* = 50) Closely Resembles the Proposed Eutherian Ancestral Karyotype (2*n* = 48) (A) G-banded karyotype of a male C. hoffmannii (2*n* = 50) with the corresponding human chromosomes indicated to the left of each chromosome. (B) Diagram of C. hoffmannii chromosomes. (C) The proposed ancestral Eutherian karyotype (2*n* = 48) [17,21]. In (B) and (C), the corresponding human chromosomes are each represented by a different color, and their numbers are indicated at the bottom of each chromosome. The chromosomes are drawn only roughly to scale. In (C), the corresponding C. hoffmannii chromosomes are indicated to the left of each ancestral chromosome. The asterisks and lines show the chromosomes that differ between the karyotypes of C. hoffmanii (CHO) and those proposed as ancestral (ANC) for Eutheria (CHO 14 and ANC 10q and 10p [black asterisks]; CHO 13 and 19 and ANC 8q [green asterisks]; and CHO 20 and 21 and ANC 7b/16p [red asterisks]).

It is interesting to note that some chromosome regions of the three Xenarthra species were not labeled by any of the human probes (Figures 2 and 3A). These regions, which were either proximal (for instance D. novemcinctus Chromosomes 25 and 30), distal (T. tetradactyla Chromosome 1 and C. hoffmanii Chromosome 7), or encompassed whole short arms (as with D. novemcinctus Chromosomes 2 and 4, T. tetradactyla Chromosome 12, and C. hoffmanii Chromosomes 3, 4, and 5), are very likely to represent repetitive sequences that are absent from the human genome.

## Discussion

The effort to establish the ancestral Eutherian karyotype relies on the concept of parsimony, according to which likely ancestral chromosomes are present in species of divergent Eutherian orders. There is a conspicuous lack of data from Xenarthra, one of the most basal mammalian groups, in the quest to establish the ancestral karyotype. Aiming to fill this gap, we conducted painting experiments with human chromosome–specific probes in species representing three of the four major Xenarthra groups.

Richard et al. [18,23] previously reported results for ten human chromosomes (3, 7, 12, 14, 15, 16, 18, 21, 22, and the X chromosome) in D. novemcinctus metaphases. The armadillo chromosomes labeled were not specified, and three of the probes (human Chromosomes 7, 12, and 22) gave results that were questioned by Richard et al. In the three cases, only one signal was observed, while the same probes labeled two (human Chromosomes 7 and 22) or three (human Chromosome 12) autosomes in our sample. We obtained the same number of signals already reported for human Chromosomes 3, 14, 15, 16, 18, and the X chromosome [18], but we detected two signals for human Chromosome 21, instead of the single labeling described by Richard et al. [18]. Human Chromosome 1 was previously shown to be conserved in C. hoffmanii [17], and this was confirmed in our experiments.

Six human chromosome associations (human Chromosomes 3/21, 4/8, 7/16, 12/22 twice, 14/15, and 16/19) are agreed, by most authors, as having been present in the ancestral Eutherian karyotype. All of these syntenies were found in the three species of Xenarthra that we studied (Table 1). The associations human Chromosomes 3/21 and 14/15 were each disrupted in, respectively, D. novemcinctus and T. tetradactyla (Figure 1F), suggesting that they underwent rearrangements in these species after the split from a common ancestor. In *C. hoffmanii,* no other syntenies besides the six already mentioned were found, and in *D. novemcinctus,* a single extra combination (human Chromosome 10/12) was identified. Human Chromosomes 9, 13, 17, 18, and 20 were found to be conserved in toto in the three Xenarthra species.

Although Xenarthra is widely accepted as a monophyletic group on morphological and molecular grounds, no common chromosome syntenies besides those considered present in the common Eutherian ancestral karyotype were found in the three species studied. Thus, there is an unusual absence of chromosome synapomorphic traits linking the three families that we studied. The only exception might be human Chromosome 8, believed to be disrupted into two blocks in a common Eutherian ancestral and found as three segments in both D. novemcinctus and T. tetradactyla and possibly also in C. hoffmannii (Table 1). Additional experiments are needed to confirm this observation.

On the other hand, we observed a striking resemblance of Xenarthran complements to the proposed 2*n* = 48 ancestral Eutherian karyotype [17,21], particularly in the case of C. hoffmannii (2*n* = 50) (Figure 3B). In this species, 20 autosome pairs appear to be the same as the most recent hypothesis for the putative Eutherian ancestral karyotype with 2*n* = 48 (human Chromosomes 1, 2p, 2q, 3/21, 4/8p, 5, 6, 7a, 7b/16p, 9, 11, 12/22 twice, 13, 14/15, 16q/19q, 17, 18, 19p, and 20). The only differences are with regard to human Chromosome 10, considered as two blocks in the ancestral karyotype and kept intact in this species; human Chromosome 8q, which might be further disrupted in C. hoffmannii resulting in a third signal for this chromosome; and an additional signal for human Chromosome 16, represented by C. hoffmanii Chromosome 20 (Figure 3).

In *D. novemcinctus,* some of the putative ancestral Eutherian chromosomes are disrupted into further blocks (for instance, human Chromosomes 1, 3/21, 6, 7, and 11), which would account for its higher diploid number (2*n* = 64) in relation to an ancestral karyotype, but otherwise this chromosome complement is also very close to that considered to be the ancestral one.

T. tetradactyla presents the most rearranged complement in relation to the ancestral karyotype, and was also reported as such when compared to other Xenarthra taxa [13]. In this case, several rearrangements seem to have occurred, resulting in chromosome disruptions and new associations in relation to a common ancestral complement. This species presented two associations previously reported in Afrotheria (human Chromosomes 1/19 and 2/8). One of them, human Chromosome 1/19, was considered as a synapomorphic trait of Afrotheria [21] and was postulated as ancestral to all Eutheria by Yang et al. [19]. Nevertheless, its occurrence in only one highly rearranged primate species [24] outside Afrotheria was considered insufficient to accept it as ancestral [21]. As human Chromosome 1/19 was found only in one of the Xenarthrans herein analyzed, and it is likely to be a different association regarding the segments involved, we do not consider it as a probable ancestral Eutherian combination.

Human Chromosome 2/8 was absent in elephants, but was found in four other species of Afrotheria that were analyzed with chromosome painting [19–22]. It was thus considered a phylogenetic link between those species after the divergence of the elephant [21]. Human Chromosome 2/8 was observed only in one species of Xenarthra, and it is not clear whether the segments involved are the same as those detected in Afrotheria, which rules out this association as a common ancestral one.

An alternative putative ancestral karyotype with 2*n* = 46 was proposed, in which the combination human Chromosome 10/12/22 would be present [20,25]. Although we observed an association human Chromosome 10/12 in *D. novemcinctus,* we could not detect it in the other two species. As previously discussed [21], the ancestral status of human Chromosome 10/12/22, which seems to be widespread in Afrotheria and Carnivores, remains doubtful. Our new data on Xenarthra do not lend support to this association as an ancestral Eutherian karyotype feature, thus reinforcing the 2*n* = 48 ancestral karyotype hypothesis.

The status of Xenarthra as a basal group in the Eutherian phylogenetic tree is still disputed on molecular grounds [3]. Based on the chromosome-painting results, it is clear that no Afrotheria has a complement so close to the putative ancestral Eutherian karyotype as the one we found herein in Xenarthra. Thus, Xenarthra cannot yet be ruled out as the most basal Eutheria, and more data should be gathered on the two basal groups before a more definitive answer is reached.

A final conclusion on the ancestral Eutherian karyotype will ultimately depend on comparisons with the most suitable outgroup, represented by marsupials. Technical constraints currently prevent cross-species chromosome painting between such distantly related groups as Eutherians and marsupials.

It is important to point out that the resolution limit of interspecies chromosome painting is about 5 Mbp. Another constraint of this approach is that it cannot detect intrachromosomal rearrangements, as inversions, that result in gene-order changes. Recent comparative studies encompassing results obtained from chromosome painting, radiation hybrid maps, and whole-genome sequencing of various mammalian groups show the potential of this broad kind of study to reveal conserved genome segments in mammals as well as the breakpoints involved in chromosome repatterning at a much more detailed level [25,26]. This approach promises ultimately to allow the reconstruction of the ancestral Eutherian karyotype, as whole-genome sequencing data become available for more mammalian species, including marsupials and monotremes, in the not too distant future.

## Materials and Methods

Chromosome preparations were obtained from cultured fibroblasts of a female nine-banded armadillo D. novemcinctus (2*n* = 64), a male two-toed sloth C. hoffmanii (2*n* = 50), and a female lesser anteater T. tetradactyla (2*n* = 54). The cells from the two former species were kindly supplied by the Center for Reproduction of Endangered Species (CRES) of the San Diego Zoo, California, United States. The lesser anteater cells were purchased from ATCC (Manassas, Virginia, United States). Cells were cultivated in αDMEM with 10% fetal bovine serum at 35 °C. Routine procedures were used for chromosome preparations. GTG banding was performed as described [27] with modifications.

Human chromosome–specific probes were obtained by flow sorting, and DOP-PCR as well as the interspecific in situ hybridization experiments were performed as already described [21].

## Abbreviations

CRES: Center for Reproduction of Endangered Species
*FN*: fundamental number (number of autosomal chromosome arms)
mya: million years ago
